# Gepantes: Aspectos Clave de Una Potente Opción Terapéutica y Consideraciones en el Contexto Latinoamericano

**DOI:** 10.31083/RN38637

**Published:** 2025-06-25

**Authors:** David Ríos Patiño, Julián Cuartas Zapata, Adolfo León Vélez Aguirre

**Affiliations:** ^1^Servicio de Neurologia, Fundación Instituto Neurológico de Colombia, 050010 Medellín, Colombia; ^2^Grupo de Investigación NeuroUnal, Unidad de Neurología, Universidad Nacional de Colombia, 111321 Bogotá, Colombia; ^3^Programa de Posgrado en Neurología, Universidad CES y Universidad de Antioquia, 050022 Medellín, Colombia

**Keywords:** gepantes, migraña, péptido relacionado con el gen de la calcitonina, tratamiento, gepants, migraine, calcitonin gene-related peptide, therapeutics

## Abstract

La migraña es una enfermedad compleja desde el punto de vista fisiopatológico, de alta prevalencia e interés prioritario en salud pública. Los gepantes son una opción terapéutica específica y de alta efectividad, su reciente llegada a Latinoamérica plantea interrogantes y expectativas acordes a nuestro contexto. Se realiza una revisión narrativa de los cuatro medicamentos disponibles actualmente fuera de latinoamerica y se exponen las principales consideraciones que a nuestro criterio deben tenerse en cuenta antes y durante su implementación. La adecuada selección de pacientes y el costo de comercialización serán los factores determinantes para su consolidación como alternativa a los medicamentos tradicionales utilizados en migraña.

## Abstract (en)

Migraine is a complex condition when considered from the perspective of its pathophysiology. Moreover, it is highly prevalent and of priority interest in public health. Gepants are a highly-effective and specific therapeutic option, although their recent arrival in Latin America raises particular questions and expectations. A narrative review of the four medications available outside Latin America at this time is provided and the main considerations that should be borne in mind before and during their adoption are presented. Appropriate patient selection and marketing cost will be key determinants for their consolidation as an alternative to traditional medications used in migraine.

## 1. Introducción

La migraña es una enfermedad neurológica crónica, incapacitante, compleja desde el punto de vista fisiopatológico, con susceptibilidad genética individual e interacción dinámica poco entendida con el entorno. Su alta prevalencia y grado de discapacidad la convierten en una patología de interés prioritario en salud pública [1].

Las opciones de tratamiento farmacológico son múltiples, desde medicamentos analgésicos hasta preventivos, con el paso de los años mucho más selectivos para la enfermedad, lo cual es una característica esencial, dada la alta frecuencia de falla terapéutica relacionada con falta de adherencia y efectos adversos [2].

La American Headache Society considera a los gepantes una opción de primera línea en el tratamiento preventivo de la migraña, teniendo en cuenta la especificidad de su mecanismo de acción, impacto en los días de dolor y disminución de la probabilidad de desarrollar una enfermedad resistente o refractaria [1, 3].

En Latinoamérica, las cefaleas primarias tienen una alta prevalencia y afectan principalmente a mujeres jóvenes. Si bien hay heterogeneidad en la información disponible, la prevalencia de cefaleas crónicas, entre ellas la migraña, es mayor que en otros grupos poblaciones [4].

Los gepantes son moléculas pequeñas que se dirigen al receptor Péptido relacionado con el gen de la calcitonina (CGRP) y lo antagonizan, interrumpiendo su vía de señalización. El auge del CGRP como elemento clave en la fisiopatogenia de la migraña, implicado hasta en el 70% de los pacientes con la enfermedad [5], convierten a los gepantes en una alternativa atractiva, presumiblemente lógica, y con alta plausibilidad en la prevención y tratamiento agudo de la migraña.

## 2. Materiales y Métodos

Revisión narrativa de la literatura, se incluyeron artículos publicados en inglés o español durante los últimos 5 años (2019–2024), en las bases de datos Pubmed, Embase y Literatura Latinoamericana y del Caribe en Ciencias de la Salud (LILACS), referentes a mecanismo de acción, eficacia, seguridad y tolerabilidad de alguno de los gepantes (rimegepante, atogepante, ubrogepante, zavegepante) en el tratamiento de la migraña. El tipo de estudio debía ser uno de los siguientes: ensayo controlado aleatorio, estudio de cohorte, estudio de casos y controles, estudio retrospectivo, revisión, o metaanálisis.

## 3. Gepantes y sus Características

### 3.1 Ubrogepante

Los antagonistas del receptor CGRP que precedieron a ubrogepante: telcagepante y MK-3207, fueron retirados debido a su hepatotoxicidad. Posteriormente se desarrolló ubrogepante, una nueva molécula con modificaciones estructurales para hacerlo menos propenso a formar metabolitos reactivos que los compuestos anteriores [6, 7]. El fármaco se une competitiva y selectivamente al receptor CGRP, con una afinidad mucho mayor en comparación con los receptores humanos de amilina 1 y adrenomedulina 2 (AMY1 y AM2) [8].

Debe evitarse su uso con inhibidores enzimáticos potentes de la Citocromo P 450 3A4 (CYP3A4) y tener precaución con inhibidores moderados. La edad, sexo, raza, peso corporal y la insuficiencia renal y hepática leve a moderada no tienen efecto sobre la farmacocinética [9]. No debe usarse en pacientes con tasa de filtración glomerular (TFG) inferior de 15 mL/min/1,73 m^2^ o diálisis, entre 15–29 mL/min/1,73 m^2^ no deben recibir más de 50 mg/dosis y puede administrarse una segunda dosis de 50 mg 2 horas después de la primera. Puede utilizarse sin ajuste de dosis en insuficiencia hepática leve y moderada, y en insuficiencia hepática grave solo 50 mg [10].

Los ensayos clínicos pivotales ACHIEVE I yACHIEVE II,realizados entre 2016 y 2018 en pacientes con migraña episódica, constituyeron la base para la aprobación del fármaco. En el estudio ACHIEVE I, se incluyeron participantes de entre 18 y 75 años, quienes fueron aleatorizados según su respuesta previa al tratamiento con triptanes y el uso concomitante o no de terapias preventivas. Los participantes recibieron una dosis única de 50 mg, 100 mg o placebo, con la opción de administrar una segunda dosis a las dos horas, en caso de persistencia o recurrencia significativa de los síntomas. Se excluyeron aquellos con enfermedad cardiovascular o cerebrovascular significativa pero no quienes tenían factores de riesgo cardiovascular. El estudio mostró libertad del dolor a las 2 horas en el 19,2% (50 mg) y 21,2% (100 mg) y mejoría del síntoma asociado más molesto en el 38,6% (50 mg) y 38,7% (100 mg) [11]. En el estudio ACHIEVE II ingresaron pacientes con las mismas edades y se aleatorizaron idénticamente al ACHIEVE I, las dosis fueron de 25 mg, 50 mg o placebo, se incluyeron pacientes con factores de riesgo cardiovascular, falla a preventivos (sin especificar cuantos), y se excluyeron nuevamente personas con comorbilidades significativas o uso excesivo de analgésicos. Lo más relevante que se encontró fue la capacidad de la dosis de 25 mg para resolver el dolor, pero no el síntoma asociado más molesto. En aquellos que recibieron una segunda dosis a las 2 horas, la presentación de 50 mg fue la más efectiva vs placebo (34% vs 19%) [12, 13]. Los pacientes de ambos ensayos fueron seguidos en un estudio abierto a un año, una cuarta parte de ellos tuvo libertad de dolor a las 2 horas con dosis de 50 mg o 100 mg [14].

Un estudio multicéntrico de fase III realizado en los EE. UU (PRODROME) evaluó la eficacia, seguridad y tolerabilidad de ubrogepante 100 mg en comparación con placebo para el tratamiento agudo de la migraña episódica cuando se administró durante el pródromo. Se excluyeron participantes que tuvieran dificultad para distinguir su migraña de otros tipos de cefalea, aquellos con uso excesivo de analgésicos y quienes tuvieran exposición previa a un anticuerpo monoclonal anti-CGRP inyectable en los 3 meses anteriores al inicio del estudio, sin especificar comorbilidades importantes o su respuesta a triptanes. La ausencia de cefalea moderada o intensa dentro de las 24 horas posteriores a una dosis de ubrogepante fue mayor en comparación con placebo (*odds ratio* (OR) 2,09, *confidence interval* (CI) 95% 1,63–2,69; *p* < 0,0001), además los eventos adversos fueron similares en ambos grupos. Los autores consideraron que Ubrogepante fue eficaz y bien tolerado cuando se tomó durante el pródromo [15].

Se han realizado estudios en personas con múltiples tratamientos fallidos, comorbilidades complejas y uso simultáneo de otros medicamentos específicos para la migraña, uno de ellos en Mayo Clinic Arizona en pacientes con migraña crónica. La eficacia, definida como ausencia o mejoría de la cefalea ≥75% de todos los ataques tratados después de 2 horas, fue del 69%. A resaltar, se encontraron posibles factores predictivos de respuesta positiva a ubrogepante: migraña con aura, migraña episódica, y <5 tratamientos preventivos o agudos fallidos. También se encontró que la respuesta al tratamiento previo a un anticuerpo monoclonal contra el CGRP (anti-CGRP) y OnabotulinumtoxinA predicen la respuesta al tratamiento y la satisfacción del paciente con ubrogepante. Adicionalmente, 16 pacientes tenían antecedentes de enfermedades cardiovasculares o cerebrovasculares importantes y no se informaron eventos adversos graves en ningún paciente [16].

Un metaanálisis reciente comparó la eficacia y seguridad de 50 mg versus 100 mg diarios de ubrogepante, no hubo diferencias en términos de eficacia, sin embargo, en seguridad se encontró que los eventos adversos aumentaron con ubrogepante 100 mg (OR = 0,81; CI 95% = 0,67–0,99; *p* = 0,040) y no hubo diferencia estadísticamente significativa de eventos adversos graves [17].

El ubrogepante es más efectivo cuando el dolor es leve frente aquellos episodios de dolor moderado o intenso, con tasas significativamente más altas de ausencia de dolor y síntomas asociados como fotofobia y sonofobia a las 2 horas después de la administración [18, 19].

Los datos sobre la eficacia en el mundo real de ubrogepante como tratamiento agudo cuando se utiliza con un anti-CGRP con o sin OnabotulinumtoxinA son limitados. Un estudio observacional prospectivo (COURAGE) evaluó el alivio significativo del dolor (MPR), el retorno a la función normal (RNF), la satisfacción con el tratamiento y la optimización del tratamiento agudo de ubrogepante (50 o 100 mg) cuando se combina con un anticuerpo monoclonal dirigido contra el Péptido relacionado con el gen de la calcitonina (anti-CGRP), OnabotulinumtoxinA, o ambos. Dicho estudio concluyó que el uso de ubrogepante en el mundo real combinado con un anti-CGRP se asoció con MPR, RNF, satisfacción y optimización aguda del tratamiento [20].

Si bien se ha demostrado la seguridad del ubrogepante en diferentes ensayos clínicos, con efectos adversos relativamente claros y consistentes, información recolectada a partir del sistema de reporte de eventos adversos de la *Food and Drug Administration* (FDA), que por supuesto permite estimar su seguridad a largo plazo, evidencia algunos eventos adversos poco comunes como hemiparesia, tinnitus, dolor torácico, hiperhidrosis, y cervicalgia [21].

### 3.2 Rimegepante

*In vitro*, rimegepante antagoniza la actividad CGRP con mayor afinidad sobre su receptor en comparación con AMY1. Es el gepante con la más alta unión a proteínas plasmáticas y se metaboliza principalmente por CYP3A4 y CYP2C9, sin metabolitos clínicamente relevantes [22].

La farmacocinética de rimegepante no se ve afectada por el sexo, la edad, la raza/etnia, el peso corporal, o el deterioro leve a moderado de la función renal (no se ha estudiado en TFG <15 mL/min o en diálisis y no se recomienda su uso) o hepática. Además, no es conveniente la administración conjunta de rimegepante con inhibidores potentes de CYP3A4 e inductores potentes o moderados de CYP3A4 [22]. Es el único gepante que podría administrarse durante la lactancia [23].

La eficacia de rimegepante para el tratamiento agudo de la migraña se evaluó en cuatro ensayos de fase III aleatorizados, doble ciego, multicéntricos y controlados con placebo. Los ensayos reclutaron pacientes de 18–65 años con ≥1 año de historia de migraña, 2 a 8 ataques de migraña de intensidad moderada o grave por mes, <15 días mensuales de cefalea e inicio de la enfermedad antes de los 50 años. Se permitió el uso concomitante de preventivos orales y se tomó en cuenta la respuesta previa a triptanes, además se excluyeron personas con antecedente de abuso de psicoactivos, uso excesivo de analgésicos, alergia a medicamentos y hallazgos en el electrocardiograma o en pruebas de laboratorio que generaran preocupaciones sobre la seguridad o tolerabilidad al medicamento, algo evaluado caso a caso por los investigadores. Los pacientes fueron aleatorizados a rimegepante 75 mg o placebo y se les indicó que trataran un solo ataque de migraña de intensidad de dolor moderado a grave. Los criterios de evaluación coprimarios fueron: la ausencia de dolor y el síntoma más molesto a las 2 horas posteriores a la dosis. El análisis agrupado de las dos presentaciones disponibles en los estudios fueron consistentes con los ensayos individuales y rimegepante proporcionó una eficacia superior al placebo para el tratamiento agudo de la migraña con alivio del dolor temprano (≤2 horas), sostenido (2–48 horas) y el retorno a la funcionalidad normal, la ausencia de náusea a las 2 horas posteriores a la dosis y la reducción del uso de medicación de rescate a las 2–24 horas posteriores a la dosis [23, 24, 25, 26].

Adicionalmente, se publicó un estudio de seguridad a largo plazo, abierto y multicéntrico para el tratamiento agudo de la migraña. Incluyó 1800 adultos divididos en tres grupos: los dos primeros con personas que tenían ataques moderados a graves de dolor (2 a 8 y 9 a 14 ataques) por mes que fueron tratados según fuera necesario con un comprimido oral de 75 mg hasta una vez por día durante 52 semanas. El tercer grupo tenía entre 4 y 14 ataques de migraña moderados a severos por mes y recibieron un comprimido de 75 mg cada dos días, más una dosis adicional para ataques de migraña de cualquier gravedad en días de dosificación no programados durante 12 semanas. Se permitió el uso de medicamentos preventivos (si tenían una dosis estable por 3 meses) y analgésicos concomitantemente pero los triptanes estaban prohibidos en la fase de tratamiento a largo plazo. Los criterios de exclusión fueron muy similares a los referidos en los estudios ya mencionados. La mayoría de los efectos adversos fueron leves o moderados, los más frecuentes fueron: infección del tracto respiratorio superior (8,8%), nasofaringitis (6,8%) y sinusitis (5,1%), no se identificaron datos de hepatotoxicidad o cefalea por uso excesivo de medicamentos y en general el fármaco fue bien tolerado [27].

Finalmente, se realizó un ensayo de fase II/III, en pacientes mayores de 18 años con 4 a 18 ataques por mes, quienes fueron aleatorizados a recibir 75 mg de rimegepante (n = 373) o placebo (n = 374) cada dos días durante 12 semanas seguido de una fase de extensión abierta de 52 semanas. Los participantes podían usar un medicamento preventivo para la migraña, excluyendo los antagonistas del receptor CGRP y los anticuerpos monoclonales anti-CGRP, siempre que la dosis fuera estable durante al menos 3 meses antes del período de observación. Se excluyó a las personas que tuvieran historia de falla a más de dos categorías de medicamentos preventivos de migraña, antecedentes o evidencia de alguna condición médica que los expusiera a un evento adverso significativo o que interferiera con las evaluaciones de seguridad o eficacia (a criterio de los investigadores), abuso de psicoactivos en los últimos 12 meses, o si tenían resultados de pruebas de laboratorio o electrocardiograma que generaran preocupaciones sobre la seguridad o tolerabilidad. Se evidenció una reducción de los días de migraña al mes en 4,3 días en comparación con 3,5 días del placebo [28]. En la fase abierta, los participantes continuaron recibiendo rimegepant 75 mg cada dos días para el tratamiento preventivo de la migraña durante 52 semanas, y también podían tomar rimegepante 75 mg según fuera necesario (hasta una vez al día) en días de dosificación no programados para el tratamiento agudo. A mayor tiempo de semanas de exposición al fármaco hubo una reducción progresiva en días de migraña hasta alcanzar las 52 semanas de tratamiento [29].

### 3.3 Atogepante

Atogepante es un antagonista competitivo con alta afinidad, potencia y selectividad por el receptor CGRP humano. Como todos los gepantes, tiene una penetración cerebral limitada y se cree que actúa principalmente inhibiendo los receptores CGRP fuera de la barrera hematoencefálica (BHE) [30, 31, 32]. El atogepante no previene completamente la activación de los nociceptores, pero si reduce significativamente la probabilidad de respuesta temprana inducida por la onda de depresión cortical en las fibras C y la probabilidad de respuesta tardía en fibras A delta [33].

No se recomienda ajuste de dosis en pacientes con insuficiencia hepática o renal leve a moderada, y se sugiere la dosis más baja de 10 mg en pacientes con insuficiencia renal grave. Los pacientes con insuficiencia hepática grave deben evitar el uso del medicamento [30]. El atogepante no genera una elevación clínicamente relevante de transaminasas y su efecto en la farmacocinética de anticonceptivos orales es mínimo [34, 35].

El estudio ADVANCE incluyó pacientes de 18–80 años con migraña episódica, aleatorizados para recibir dosis de 10–30–60 mg del medicamento o placebo durante 12 semanas. Se excluyeron participantes con posibles neuropatías craneales dolorosas, cefalea diaria persistente de novo o alguna cefalalgia trigémino autonómica, quienes hubieran tenido una respuesta inadecuada a más de cuatro medicamentos preventivos orales y quienes cursaran con uso excesivo de analgésicos. Se analizaron los datos de 873 pacientes y se evidenció disminución en los días de migraña al mes: 1,2 días (10 mg), 1,4 días (30 mg), y 1,7 días (60 mg) vs placebo; además, también mejoraron los días de cefalea/mes y los desenlaces de calidad de vida [36].

Entre los años 2018–2020 un estudio multicéntrico realizado en Estados Unidos evaluó la eficacia, seguridad y tolerabilidad de 60 mg de atogepante vs placebo durante 1 año. Se excluyeron personas con migraña crónica, dificultad para diferenciar su migraña de otras cefaleas, falla a tres medicamentos preventivos orales, uso excesivo de analgésicos o alteraciones relevantes en exámenes de laboratorio a criterio de los investigadores. La proporción de participantes con reducciones ≥50%, ≥75% y 100% en días de migraña/mes aumentó del 60,4% (310/513), 37,2% (191/513) y 20,7% (106/513) en la semana 1–4, a 84,2% (282/335), 69,9% (234/335) y 48,4% (162/335), en las semanas 49–52, además se encontró un buen perfil de seguridad y tolerabilidad [37].

El estudio PROGRESS evaluó la eficacia, tolerabilidad y seguridad del atogepante en personas de 18–80 años con migraña crónica en 148 instituciones de Norteamérica, Europa y Asia. Fueron excluidos aquellos participantes con antecedentes de migraña acompañada de diplopía o disminución del nivel de conciencia, migraña retiniana, diagnóstico de cefalea diaria persistente de novo, cefalalgias trigémino autonómicas, respuesta inadecuada a más de cuatro tratamientos preventivos orales, así como el uso de opioides o barbitúricos durante al menos 4 días al mes en los 3 meses previos al inicio del estudio. Se excluyeron además participantes con cualquier enfermedad clínicamente significativa según los investigadores. La duración del estudio fue de 12 semanas y la intervención fue 60 mg una vez al día vs 30 mg dos veces al día vs placebo. Los autores utilizaron la dosis más alta del medicamento teniendo en cuenta el estado avanzado de la enfermedad y la mayor discapacidad que confiere frente a la migraña episódica. La reducción en días de migraña/mes vs placebo fue de—2,4 días con 30 mg dos veces al día y—1,8 con 60 mg una vez al día. Nuevamente el perfil de seguridad y tolerabilidad fue bastante bueno [38].

También se realizó un análisis comparativo a partir de los datos de los estudios ADVANCE y PROGRESS y el ensayo BHV3000-305 con rimegepante, para evaluar la eficacia y la seguridad/tolerabilidad de ambos como preventivos. Los participantes que recibieron 60 mg de atogepante una vez al día, rimegepante en tabletas orodispersables por vía oral de 75 mg (interdiario) y placebo. Hubo 252 participantes en el grupo de atogepante y 348 en el grupo de rimegepante. Durante las semanas 1 a 12, atogepante 60 mg demostró una mayor reducción en los días mensuales de migraña en comparación con rimegepante 75 mg (CI del 95%: –1,65 –2,49, –0,81; *p* < 0,001) [39].

El ensayo ELEVATE evaluó la seguridad, tolerabilidad y eficacia de atogepante para el tratamiento preventivo de la migraña episódica en personas con falla a 2–4 clases de tratamientos preventivos orales convencionales, sin considerar contraindicación como un equivalente a falla terapéutica. Cualquier medicamento con eficacia conocida para la prevención de migraña, incluidos los tratamientos preventivos orales estaban prohibidos, asi como los anticuerpos monoclonales dirigidos contra el CGRP y la toxina botulínica, también se excluyeron personas con uso excesivo de analgésicos. Fue un ensayo de fase IIIb, multicéntrico, realizado en Canadá, Estados Unidos y varios países europeos. Los brazos del estudio fueron 60 mg de atogepante o placebo y el desenlace principal fue la reducción en días mensuales de migraña, con un desenlace primario favorable al atogepante [40].

La combinación de atogepante y OnabotulinumtoxinA puede tener un efecto sinérgico en pacientes con Migraña crónica, debido a sus distintos mecanismos de acción (en fibras A delta y C respectivamente). Los estudios en modelos animales han demostrado que su uso combinado puede prevenir la activación y sensibilización de las neuronas trigéminovasculares nociceptivas en el núcleo espinal del trigémino inducida por la depresión cortical propagada [41].

### 3.4 Zavegepante

*In vitro*, zavegepante se metaboliza predominantemente por la CYP3A4 y en menor medida, por CYP2D6. La edad, el sexo, la raza, el peso corporal y la insuficiencia hepática leve y moderada (Child-Pugh A y B), no afectan la farmacocinética del medicamento y no se ha evaluado en insuficiencia hepática o renal grave [42]. Zavegepante fue aprobado por la FDA en el 2023 en base a un estudio fase II/III y otro fase III. En ambos ensayos se incluyeron pacientes con migraña episódica, con al menos un año de enfermedad y se permitió el uso de medicamentos preventivos, no se excluyeron quienes tuvieran contraindicación a triptanes siempre y cuando cumplieran con todos los demás criterios de inclusión. Los antagonistas del CGRP debían haberse suspendido al menos 6 meses antes de la visita de selección y estaba prohibidos durante el estudio, así como los opioides y barbitúricos, también fueron excluidas personas con migraña con aura del tronco encefálico, migraña hemipléjica y condiciones médicas significativas a criterio de los investigadores, además no se permitió el uso de aerosoles nasales de cualquier tipo. La dosis efectiva utilizada en los estudios fue de 10–20 mg intranasal, superando al placebo en cuanto al alivio del dolor a 2 horas y mejoría del síntoma acompañante más significativo para el paciente. Los efectos adversos más frecuentes fueron disgeusia, disconfort nasal y náusea. La efectividad del zavegepante versus analgésicos convencionales ya ha sido analizada en revisiones sistemáticas y metaanálisis con resultados favorables [43] y se ha postulado un alivio del dolor más rápido frente a los otros gepantes (en 15 minutos) pero se requieren más estudios [44, 45, 46].

## 4. Discusión

La alta unión a proteínas, metabolismo predominantemente hepático, precaución con inhibidores e inductores enzimáticos de la CYP3A4, tiempo de vida media corto, y alta lipofilicidad, son las principales características farmacocinéticas de los gepantes (Tabla 1, Ref. [4, 13, 16, 23, 29, 30, 34, 47]).

**Tabla 1.** **Perfil de seguridad de los gepantes [4, 13, 16, 23, 29, 30, 34, 47]**.

Gepante	Indicación	Efectos adversos 12 semanas	Efectos adversos frecuentes a 52 semanas	NNT (Número necesario a tratar)	Uso en gestantes y niños	Uso en lactancia	Riesgo cardiovascular
Ubrogepante	Tratamiento agudo	Náusea 1,9%, Mareo:1,2%	Náusea 1,7–4%	25 mg: 8	Sin evidencia	No hay evidencia concluyente	Sin evidencia de efectos adversos
		Somnolencia: 0,7%	Somnolencia: 0,6–2,5%	50 mg: 12			
		Boca seca: 0,4%	Boca seca: 2,6–2,1	100 mg: 5			
Atogepante	Tratamiento preventivo	Constipación: 6,9–7,7%	Infección respiratoria superior: 10%	10 mg: 3,8	Sin evidencia	Sin evidencia	Sin evidencia de efectos adversos
		Infección respiratoria superior: 3,9–5,7%	Constipación: 7%	30 mg: 3,4			
		Náusea: 6,1–4,4%	Náusea: 6%	60 mg: 3,1–4,2			
Rimegepante	Tratamiento agudo y preventivo	Nasofaringitis: 4%	Nasofaringitis : 6,8%	75 mg: 8	Sin evidencia	Compatible	Sin evidencia de efectos adversos
		Náusea: 3%	Sinusitis: 5,1%				
		Infección urinaria: 2%	Infección urinaria: 3,8%				
Zavegepante	Tratamiento agudo	Disgeusia: 21%	**-**	10 mg: 9,6–12	Sin evidencia	Sin evidencia	Sin evidencia de efectos adversos
		Disconfort nasal:4%					
		Náusea: 3%					

La evidencia de los gepantes aún se está construyendo, sin embargo, en una revisión realizada por nuestro grupo académico asistencial en cefaleas, nos ha sido posible reconocer ventajas sobre otras opciones terapéuticas que ayudan a perfilar el tipo de paciente que más se beneficiaría: aquellos con uso excesivo de analgésicos, condición no asociada a los gepantes; mujeres en edad reproductiva, que tienen una posibilidad de rápida suspensión del medicamento si hay gestación, ya que la corta vida media lo permite, además el uso de rimegepante ha demostrado ser seguro durante la lactancia; personas con factores de riesgo cardiovascular y quienes deseen solo medicamentos vía oral [19, 48] . Además, los efectos adversos más comunes generalmente no constituyen una gran limitación para la implementación del tratamiento, siempre y cuando se realice una adecuada caracterización de los síntomas más molestos para el paciente y se le brinde una explicación detallada sobre los efectos adversos más probables. El beneficio en los síntomas acompañantes al dolor, que muchas veces resultan ser los más incapacitantes (como la fotofobia o la náusea), y la posibilidad de combinación con otras alternativas de tratamiento como la OnabotulinumtoxinA, son elementos que también inclinan la balanza hacia el uso de los gepantes.

Los gepantes son una novedosa opción terapéutica para un grupo poblacional cuya enfermedad es dependiente del ya muy reconocido CGRP; los estudios pivotales, así como algunos estudios de la vida real ya certifican su seguridad (Tabla 2, Ref. [6, 10, 23, 31]), pero para el contexto de Latinoamérica, por la diversidad étnica y el mestizaje, se debe explorar tanto su efectividad como su tolerancia. Es así como la expectativa respecto a estos medicamentos, amplía el panorama en lo correspondiente a opciones terapéuticas de última generación en el tratamiento de la migraña.

**Tabla 2.** **Aspectos farmacocinéticos más relevantes de los gepantes [6, 10, 23, 31]**.

Gepante	Presentación	Ruta de administración	Dosis/dosis máxima	Ajuste de dosis	Unión a proteínas	Tmax	Vida media	Metabolismo	Eliminación
Ubrogepante	Comprimidos 50–100 mg	Oral	50–100 mg (segunda dosis opcional a las 2 horas de la inicial)/200 mg en 24 horas	TFG <30 mL/min, Child–Pugh C.	87%	1,5 horas	5–7 horas	CYP3A4	Biliar> renal
				No usar si TFG <15 mL/min					
Atogepante	Comprimidos 10 mg, 30 mg, 60 mg	Oral	10–60 mg/60 mg en 24 horas	TFG <30 mL/min, Child–Pugh C	95%	2 horas	11 horas	CYP3A4	Biliar> renal
				No usar si TFG <15 mL/min					
Rimegepante	Comprimidos y presentación	Oral	75 mg/75 mg en 24 horas	TFG <15 mL/min, Child–Pugh C	96%	1,5 horas	11 horas	CYP3A4>CYP2C9	Biliar> renal
	Orodispersable 75 mg								
Zavegepante	Inhalador nasal	Intranasal	10 mg/10 mg en 24 horas	No usar si TFG <15 mL/min	90%	30 minutos	6,5 horas	CYP3A4>CYP2D6	Biliar> renal
	10 mg/puff								

TFG, tasa de filtración glomerular.

Las anteriores consideraciones se hacen reconociendo que los ensayos clínicos de migraña muestran una gran variabilidad en los resultados y criterios de evaluación de desenlaces utilizados, a pesar de los esfuerzos de estandarización planteados por entidades como la Sociedad Internacional de Cefaleas [47].

En Latinoamérica hay falta de datos sobre prevalencia de migraña en muchos países y se dispone de información recolectada a partir de estudios descriptivos que datan del milenio pasado. Todo esto facilita heterogeneidad en las estimaciones: si bien las cifras de prevalencia global y ajustada a un año son similares entre los estudios realizados en Latinoamérica y España (la mayoría entre 12,4% y 19,6%) algunos muestran prevalencias muy elevadas, (29,5% Brasil) y otras muy bajas (7,12% Colombia) [49].

Por otro lado, no disponemos de investigaciones en Latinoamérica que estudien el impacto socioeconómico de la migraña, mientras en otros países como Estados Unidos se considera un problema de salud pública, dada la carga a los sistemas de salud, impacto en la productividad laboral y alta prevalencia en la población más vulnerable [50].

El uso de los gepantes requiere una adecuada selección de los pacientes, dado que no solo incluye variables clínicas, sino también económicas. A la fecha de elaboración de éste manuscrito, solo se ha publicado un estudio de costoefectividad, para atogepante y rimegepante y ninguno de los medicamentos pudo alcanzar los umbrales de rentabilidad generalmente aceptados (<1500.000/años ajustados de calidad de vida) en Estados Unidos, por supuesto se necesitan estudios adicionales para guiar una mejor la toma de decisiones, no solo en Estados Unidos y Europa en los que se centra el mercado, sino también en Latinoamérica [51]. Además, existe una realidad geopolítica postpandemia que contribuye a considerar la potencial prescripción de este tipo de medicamentos, en lo que se refiere a las barreras administrativas, tanto de tipo gubernamental (inclusión en los planes de beneficio de aseguradores) como de capacidad económica de la población para adquirirlos.

## 5. Conclusiones

La evidencia demuestra que los gepantes tienen un buen perfil de seguridad hepática y alta probabilidad de adherencia por sus presentaciones y seguridad cardiovascular. Consideramos que son una herramienta terapéutica potente y específica para pacientes con diagnóstico de migraña, pero en casos adecuadamente seleccionados. Se espera que en Latinoamérica se logre el acceso, implementación y posterior consolidación como alternativa a los medicamentos tradicionales utilizados en migraña. El tiempo y su utilización en el ámbito clínico Latinoamericano nos mostrarán la realidad.

## Data Availability

Los datos y materiales utilizados en este estudio se describen en la sección de Materiales y Métodos. La información está disponible en las bases de datos EMBASE, LILACS y PUBMED.

## Contribuciones de los Autores

DRP, JCZ y ALVZ participaron en el diseño del estudio, la recolección y análisis de la información, la supervisión metodológica, la redacción y revisión crítica del manuscrito. Todos los autores leyeron y aprobaron el manuscrito final. Todos los autores han participado lo suficiente en el trabajo y han acordado ser responsables de todos los aspectos del mismo.

## Aprobación Ética y Consentimiento Informado

No aplicable.

## Agradecimientos

No aplicable.

## Financiación

Esta investigación no recibió financiación externa.
